# Effects of Postpartum Supplemental Oral Ca for Dairy Cows Fed Prepartum Dietary Acidogenic Salts

**DOI:** 10.3390/ani11113131

**Published:** 2021-11-02

**Authors:** David B. Vagnoni, Michayla Davidson, Livia Rubio, Garrett R. Oetzel, Emmanuelle Comets

**Affiliations:** 1 Department of Animal Science, California Polytechnic State University, San Luis Obispo, CA 93407, USA; michdavidson2@gmail.com (M.D.); liv.rubio27@gmail.com (L.R.); 2 School of Veterinary Medicine, University of Wisconsin, Madison, WI 53706, USA; gary.oetzel@wisc.edu; 3 INSERM, IAME, Université de Paris, F-75006 Paris, France; emmanuelle.comets@inserm.fr; 4 INSERM, Université Rennes-1, CIC 1414, F-35700 Rennes, France

**Keywords:** acidogenic salts, oral calcium, hypocalcemia, milk fever

## Abstract

**Simple Summary:**

Low blood calcium after calving is problematic in dairy cows, particularly for older cows and for Jerseys vs. Holsteins. Feeding acidogenic salts before calving and oral Ca supplementation post-calving help mitigate this. We evaluated: (1) a novel approach to delivering acidogenic salts prepartum applicable to small dairies and (2) the combined effects of prepartum acidogenic salts and postpartum oral Ca supplementation for both Holsteins and Jerseys of varying parity. We found this novel approach to providing acidogenic salts to be effective. We also found preliminary evidence that shortening the time of application of this technology may enhance its beneficial effects. Responses to prepartum acidogenic salts and postpartum oral calcium supplementation were very different for second vs. greater than second parity cows. Finally, we found an increased risk of low blood calcium, particularly in older Jersey cows, when either prepartum urinary calcium excretion was low or colostrum production was high.

**Abstract:**

Postpartum hypocalcemia is a problem in dairy cows. Both the Jersey vs. Holstein breed and increasing parity are known risk factors. Our objectives were: (1) to evaluate a simple approach to provide dietary acidogenic salts suitable for application on small dairies and (2) to evaluate the combined effects of degree of acidification and oral Ca supplementation along with breed and parity group on periparturient Ca status of Holstein and Jersey cows. Cows were moved weekly from the far-off dry pen at 260 days pregnant to the close-up pen, where all cows received the acidogenic diets. The diet was offered as a total mixed ration and CaCl_2_, and our source of acidogenic salts was top-dressed in liquid form and mixed in by hand. Thirty-six cows were blocked by parity group (parity = 2 vs. parity ≥ 3) and breed (Holstein vs. Jersey) and assigned to one of two treatments (no intervention or postpartum oral Ca bolus supplementation) in an alternating fashion, based on expected date of parturition. Urinary acidification appeared complete within 3–4 days. Increased urinary Ca excretion was >93% of maximum from 7–21 days before falling to <5% of maximum by 28 days. Serum Ca concentrations 12–24 h postpartum were lower for Jerseys vs. Holsteins and for parity ≥ 3 vs. parity = 2 cows. Serum Ca over 6–48 h postpartum decreased and increased, respectively, with oral Ca supplementation for parity = 2 and parity ≥ 3 cows. Decreased prepartum urinary Ca excretion and increased colostrum yield appear to be independent risk factors of hypocalcemia for parity ≥ 3 Jerseys.

## 1. Introduction

Due to the challenge of Ca homeostasis imposed by the sudden Ca demand from the onset of colostrogenesis and subsequent lactation, subclinical and clinical hypocalcemia have been estimated to be approximately 50% and 5% of multiparous dairy cows in the U.S., respectively [1]. The total cost per case of hypocalcemia has been estimated to be $246 [2]. Hypocalcemia may result in additional losses because it is a gateway disease [3] that increases the risk for other parturient metabolic disorders by impairing gastro-intestinal motility, delaying teat sphincter closure, reducing dry matter (DM) intake, impairing immune function, and increasing the risk for herd removal [4]. Risk factors for hypocalcemia include excessive body condition, increasing parity, increased dietary cations relative to anions [5] and being of the Jersey rather than the Holstein breed [6].

Lowering the dietary cation anion difference (DCAD), which is typically computed as the difference (meq/kg DM) of (Na + K) − (Cl + S), induces a compensated metabolic acidosis that is characterized by reductions in blood bicarbonate, base excess, and pH along with increased urinary net acid excretion and decreased urinary pH [7]. This strategy is commonly employed during the last 21 days prepartum to reduce the risk of milk fever [8]. The mode of action of this compensated acidosis is believed to be a result of a release of bone Ca to maintain blood pH [7] and enhanced tissue receptivity to parathyroid hormone [3], which facilitates this bone resorption, activates vitamin D, and increases active intestinal Ca absorption.

Dairy cows increased urinary Ca excretion 6-fold [9] or 20-fold [10] when presented with excess Ca by means of either intravenous infusion or administration of vitamin D metabolites, respectively. Thus, increased urinary disposal of Ca is an important mechanism for cows to maintain normocalcemia when Ca supply greatly exceeds Ca requirements. Feeding low DCAD diets prepartum presents the same challenge. Namely, the induced bone Ca mobilization without a concomitant increase in Ca requirements presents the cow with a large excess Ca supply. The resultant large increases in urinary Ca excretion [11,12] likely represent a protective mechanism against hypercalcemia. This Ca that is mobilized and excreted prior to calving can be conserved and used by the cow when Ca is demanded [13].

Reducing DCAD may be accomplished using a variety of commercial products or by the use of acidogenic salts of chloride or sulfate (e.g., CaCl_2_ or MgSO_4_). Cows’ aversion to these salts [14] is frequently ascribed to unpalatability but likely is a response to the resultant metabolic acidosis [11,15]. In either case, proper mixing of dietary anion sources into the ration is critical to ensure proper intake. Proper application of DCAD technology is challenging for small farms, which typically cannot mix a separate total mixed ration (TMR) for the close-up dry cow pen because the mixer batch size is too small.

Another strategy for mitigating milk fever is providing supplemental oral Ca immediately postpartum. Different commercial preparations, containing a variety of Ca salts, have been demonstrated to increase serum Ca concentrations immediately postpartum [16,17,18]. Milk production responses to supplemental oral Ca include an interaction whereby production numerically increased in high producing cows but numerically decreased in low producing cows [19,20] or no effect [21]. When the Ca is in the form of CaCl_2_ or CaSO_4_ (i.e., acidogenic salts), the associated acidogenic effect [16,22,23] may be additionally beneficial. All of the above studies employed reduced DCAD; responses to supplemental oral Ca were observed while feeding acidogenic salts.

Based on the above, the current study was undertaken with several objectives in mind pertaining to cows receiving acidogenic salts. Our first objective was to evaluate the effectiveness of a simple approach to lowering DCAD in the close-up dry cow group by applying a commercially available CaCl_2_ solution as a top dress and hand-mixing it with the TMR. The second study objective was to utilize natural variation among cows to evaluate the continuous variables prepartum urinary pH, prepartum urinary Ca excretion, and colostrum production on postpartum serum Ca concentrations. The third objective was to evaluate the effects of the categorical variables breed (Holstein vs. Jersey) and parity group (parity = 2 vs. parity ≥ 3), oral Ca supplementation, and interactions among these categorical and the continuous covariates on postpartum serum Ca concentrations.

## 2. Materials and Methods

### 2.1. Animals, Diets, and Samples

This study was conducted from November 2019 to March 2020 at the California Polytechnic State University (San Luis Obispo, CA, USA) dairy unit. All animal use and procedures were approved by the Institutional Animal Care and Use Committee of the university. Cows (*n* = 36) were blocked by parity group (parity = 2, *n* = 17 or parity ≥ 3, *n* = 19) and breed (Holstein, *n* = 15 or Jersey, *n* = 21) and then assigned to one of two treatment groups in an alternating fashion, based on expected date of parturition (Supplemental Table S1). Treatments consisted of either a control group (CON) receiving no intervention after parturition or a group receiving two doses of 43 g of elemental Ca as a Ca oral supplement (CaOS) via bolus (Bovikalc, Boehringer Ingelheim, St. Joseph, MO, USA) 12 h apart.

Cows were moved weekly from the far-off dry cow pen at 260 days (±2.8 SD) pregnant to the close-up dry cow pen. Urine samples were obtained the evening prior to this move and then every 3–4 days thereafter at 7:00 a.m. until parturition. Urine samples were collected mid-stream by manually stimulating the area immediately ventral to the vulva. In the close-up pen, all animals were housed together in a single pen with 77 cm wide headlocks; stocking density (i.e., cows/headlocks) never exceeded 1. Cows in the close-up dry cow pen were fed the same TMR as in the far-off dry cow pen (Supplemental Table S2). Cows were fed once daily at 3:00 p.m. to provide a target of 10% refusals. Immediately after feed delivery, a liquid calcium chloride solution (CaCl_2_; 32% solution (*wt*/*wt*), Nelson-Jameson, Marshfield, WI, USA) was provided to lower the DCAD. This solution was applied via a sprinkler can and was manually mixed into the TMR in the bunk using a pitchfork.

Urinary pH values were determined immediately upon collection using a calibrated electronic pH meter. The goal was to maintain mean urinary pH values within the range of 6–7. As a group, mean pH values approached the lower or upper range, and the daily CaCl_2_ dose was adjusted up or down, respectively, in increments of about 0.25 equivalents Cl/cow.

Cows were moved to a separate pen within 0 to 3 h following parturition. They were placed in a headlock, milked, the weight of colostrum produced was recorded, and a blood sample was obtained from the coccygeal artery or vein into a serum tube. Cows in the CaOS group then received one bolus and then again after 11.5 h (±3.7 SD). Additional blood samples were obtained from all cows at 0.5, 1, 6, 12 (immediately prior to the second bolus), 24, 48, 72, 96, and 168 h relative to administration of the first Ca bolus. Blood samples were refrigerated immediately upon collection, collected twice weekly, and centrifuged at 2000× *g* for 10 min. The serum was separated and frozen at −20 °C for subsequent Ca analysis. Whole-blood beta-hydroxybutyrate (BHB) was measured cowside at the 96 and 168 h sampling using the BHBCheck Plus System (PortaCheck, Moorestown, NJ, USA).

Urine samples were centrifuged (2000× *g*, 5 min), acidified with 6N HCl to pH < 3, and stored frozen. Samples of fresh feed were obtained weekly, immediately prior to administration of CaCl_2_, and samples of orts were obtained the next day immediately prior to removal. Subsamples of dried (60 °C for 48 h) feed and orts as well as weekly samples of CaCl_2_ solution were stored at room temperature until the completion of the trial. Weekly samples of feed and orts were ground (1-mm screen, Wiley mill) and then composited into biweekly samples (*n* = 9). Weekly CaCl_2_ samples were similarly composited and then all samples were sent to Cumberland Valley Analytical Services (Waynesboro, PA, USA) for nutrient analyses.

Calcium concentration in urine and serum was determined using a commercial kit (Pointe Scientific C7529500, Fisher Scientific, Waltham, MA, USA). Urinary creatinine concentration was determined [24], and the urinary Ca:creatinine ratio was computed to correct for dilution due to differing urinary volumes among samples. Our inter-assay coefficients of variation for urinary and serum Ca concentrations were 6% and 5%, respectively.

### 2.2. Statistical Analyses

#### 2.2.1. Prepartum Urinary pH

The primary purpose of measuring pH was to ensure appropriate acidification (deemed as values within the range of 6 to 7) and to serve as a covariate for other outcomes of interest. Overall mean urinary pH (UpH) of all urine samples collected after commencement of acidification until parturition was computed by cow and used as a covariate for the prepartum urinary Ca:creatinine ratio analysis (detailed below). Mean urinary pH from the last 3 samples prepartum was used as a proxy for urinary pH at day of calving (UpHdoc) and used as a covariate for postpartum data (i.e., blood BHB and serum Ca concentrations). Statistical analyses revealed that sample number was not significant (*p* = 0.48) among these last 3 samples, confirming the appropriateness of this approach.

#### 2.2.2. Prepartum Urinary Ca Excretion

Plots of prepartum urinary Ca excretion (UCE), expressed as Ca:creatinine ratio (mol:mol), suggested a sigmoidal increase and subsequent decrease over time (Supplemental Figure S1. Various double sigmoidal functions, accommodating an ascending and subsequent descending phase were constructed, including an exponential function with a discrete lag, logistic function, and Gompertz function. Fits to these functions were evaluated based on information criteria, robustness, and biological interpretability of the parameters. Based on the above criteria, the final model selected was the logistic equation based on the parameterization of Zwietering et al. [25]. Model parameterization consisted of the baseline values prior to acidification (BL), the maximum increase above baseline (A), the maximal rate (days^−1^) of increase (μ_1_) and decrease (μ_2_) and the corresponding time (days) of maximal rate of increase (λ_1_) and decrease (λ_2_) in Equation (1) below:

(1) $$f\left(t\right)=\mathrm{BL}+\frac{A}{1+\exp\left[\frac{4\mu_{1}}{A}\left(\lambda_{1}-t\right)+2\right]}\times \frac{1}{1+\exp\left[\frac{4\mu_{2}}{1}\left(t-\lambda_{2}\right)+2\right]}$$

To fit this function, we used a nonlinear mixed effects model (NLMEM), using the *saemix* package of R [26]. Denoting i as the individual cow (I = 1, …, N), the following statistical model was assumed to describe the observation y_ij_ at time t_ij_:

y_ij_ = f(t_ij_, φ_i_) + g(t_ij_, φ_i_) ε_ij_ (2)

where f is the structural model from Equation (1), φ_i_ represents the parameters for cow i (φ_i_ = (BL_i_, A_i_, μ_1i_, μ_2i_, λ_1i_, λ_2i_)), and ε_ij_ is a residual error assumed to follow a standard normal distribution, with g representing the variance of the observations. The distribution of each individual parameter in the population was assumed to follow a log-normal distribution, with the joint distribution of the random effect modelled as a multivariate normal with covariance matrix Ω.

Individual parameters were estimated using this base model as the mean of the conditional distribution, and significant (*p* ≤ 0.05) covariates were determined using the method in Xu et al. [27]. Categorical covariates evaluated consisted of breed, parity group, and their interaction while continuous covariates evaluated consisted of the centered mean values (by cow) of UpH and equivalents of Cl offered per day during the time of acidification. Based on this final model and the number of days of acidification prior to calving, the modeled urinary Ca excretion at day of calving (UCEdoc) was computed for each cow. This variable was evaluated as a covariate for subsequent analysis of the postpartum serum Ca data.

#### 2.2.3. Postpartum Time Course of Serum Ca and BHB

Time course postpartum serum Ca and BHB data were fit to a linear mixed model using the lme function of the nlme package [28] to evaluate the effects of time and its interactions with covariates. Categorical covariates consisted of breed, parity group, treatment, and sampling time. Centered continuous covariates consisted of days of acidification, mean daily equivalents of Cl over the last 3 samples prepartum, UpHdoc, UCEdoc, and colostrum production (kg). Data were fit to the full model which contained the main effects and all possible interactions among categorical covariates as well as the linear and quadratic terms of each continuous covariate (interactions between categorical and continuous covariates were not accommodated by the model). The model also included individual cow as a random effect and autocorrelation was accommodated using a first order autoregressive function, *corAR*(1). After fitting the full model, backwards elimination was carried out until only significant (*p* ≤ 0.05) terms remained (non-significant terms involved in significant interactions were always retained). When there was a significant (*p* ≤ 0.05) interaction among categorical covariates and time, least squares means corresponding to the interaction were compared using a Bonferroni correction for the number of time points compared.

#### 2.2.4. Postpartum Univariate Measures: Serum Ca Concentration, UCEdoc and Colostrum Production

Two univariate measures of serum Ca concentrations were evaluated, the value immediately post-calving (Ca0) and the minimum value observed over time (CaMin). These data were analyzed by generalized least squares using the *gls* function of the *nlme* package [28]. Categorical covariates consisted of breed, parity group, and supplemental oral Ca treatment while centered continuous covariates consisted of mean daily equivalents offered obtained from the last 3 samples prior to parturition, UpHdoc, UCEdoc, and colostrum production (kg). Categorical and continuous covariates were evaluated using two separate approaches. For categorical covariates, the full statistical model consisted of all main effects and interactions among the categorical covariates and the linear and quadratic terms of each centered continuous covariate (interactions between categorical and continuous covariates were not accommodated by the model). Backwards elimination was conducted as described above, and the responses of interest were the least squares means corresponding to categorical covariates. Assessment of the impact of continuous covariates was as follows. The r^2^ obtained between UCEdoc and UpHdoc was 0.31 and the r^2^ between other pairs of covariates was ≤0.12. These variables were therefore judged to be largely independent, and each continuous covariate was individually fit to a model containing the main effects and all interactions among categorical covariates and the centered continuous covariate of interest. In this instance, the terms containing the continuous covariate (i.e., either main effects or interactions) correspond to slopes of the relationship between the dependent variable and the continuous covariate. Backwards elimination was conducted as described above and the outcomes of interest consisted of: (1) the statistical significance of these terms and (2) the least squares estimates of these terms and their statistical difference from zero.

Each of UCEdoc and colostrum yield data were analyzed by generalized least squares using the *gls* function of the *nlme* package [28], where the model effects consisted of breed, parity group, and the interaction of breed with parity group.

## 3. Results

Two Jerseys (each fourth parity, group CON) were diagnosed with clinical milk fever (defined as a recumbent cow unable to stand and walk that responded to intravenous Ca gluconate) and removed for the remainder of the trial. Serum Ca concentrations in blood samples taken immediately prior to treatment were 0.80 mmol/L at 12 h for one cow and 0.90 mmol/L at 24 h for the other cow. These values confirmed the diagnosis of clinical milk fever for both cows. All data for these two cows obtained up to the time of their clinical milk fever, including their pre-treatment serum Ca concentrations, were included in the statistical analyses.

The overall mean dose of CaCl_2_ offered was 3.38 (±0.79 SD) equivalents Cl/cow per day and the overall mean UpH for cows during the close-up dry period was 6.32 (±0.46 SD). The DCAD prior to addition of CaCl_2_ solution was 134 meq/kg DM. Overall mean calculated DCAD value of the diet following CaCl_2_ addition was −85 meq/kg DM (based on the overall mean DM intake of 14.6 kg DM/head/day and equivalents of Cl offered). Actual Cl intake was calculated to be 89% of expected intake, based on Cl analyses of feed offered and refused. These results indicated that cows were modestly effective in sorting against the added Cl. Finally, the balanced nature of the experiment was confirmed because analysis of variance revealed no significant differences (*p* ≥ 0.24) among breeds, parity groups, or treatments on mean equivalents of added Cl/day, UpH, or Ca0.

### 3.1. Urinary pH and Ca Excretion

**Figure 1:**
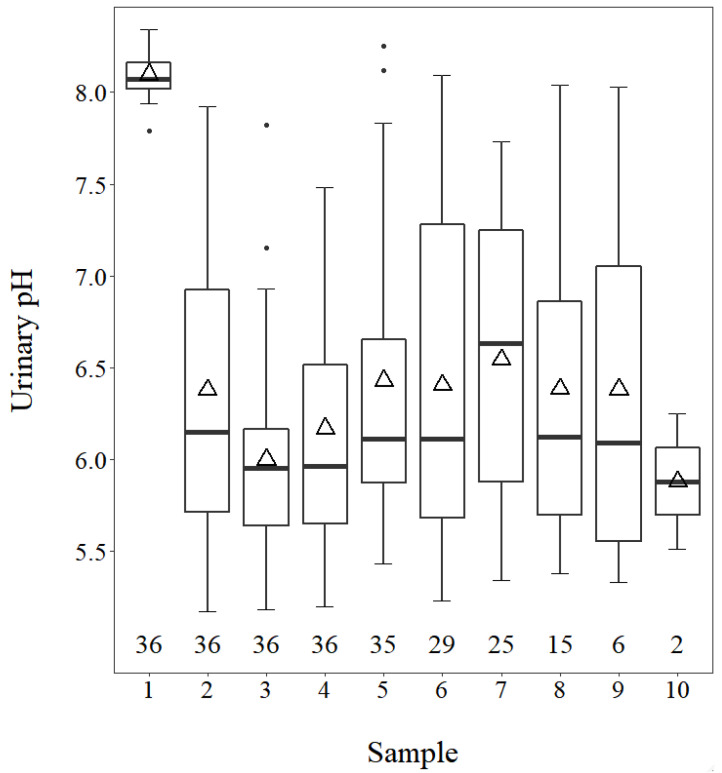
Tukey boxplot of urinary pH in samples taken 3–4 days apart prior to (sample 1) or following (samples 2–10) introduction of dietary CaCl_2_. Numbers above the *x*-axis represent the number of samples at each sampling time. Boxes delimit the interquartile range and include the median (line) and mean (∆). Upper and lower whiskers denote observations within 1.5 times the 75th and 25th percentile from the median, respectively, and dots represent data points beyond those limits.

Prior to acidification, urinary pH (Figure 1) averaged 8.14 and exhibited very little variation. Following the introduction of dietary CaCl_2_, the mean pH of all subsequent samples (taken 3–4 days apart) remained between 6 and 7, which was our goal. In addition, as expected, variation among samples was substantially greater once the cows were acidified (Figure 1).

Parameter estimates of prepartum UCE obtained from fitting Equation (1) are presented in Table 1. The maximum increase above baseline was greater for Jerseys vs. Holsteins (main effect of breed, *p* = 0.011). For perspective on the overall magnitude of this increase, maximal values of UCE relative to baseline values represented a 5.4 and 6.4-fold increase for Holsteins and Jerseys, respectively. As the parity group increased, this value numerically increased for Holsteins but numerically decreased for Jerseys (interaction of breed with parity group, *p* = 0.025). Finally, this value was increased 0.605 units per 1-unit increase in equivalents of Cl offered/day (*p* = 0.002). Maximum rates of increase and decrease in UCE occurred at 2.83 and 26.8 days following the introduction of dietary CaCl_2_, respectively.

**Figure 2:**
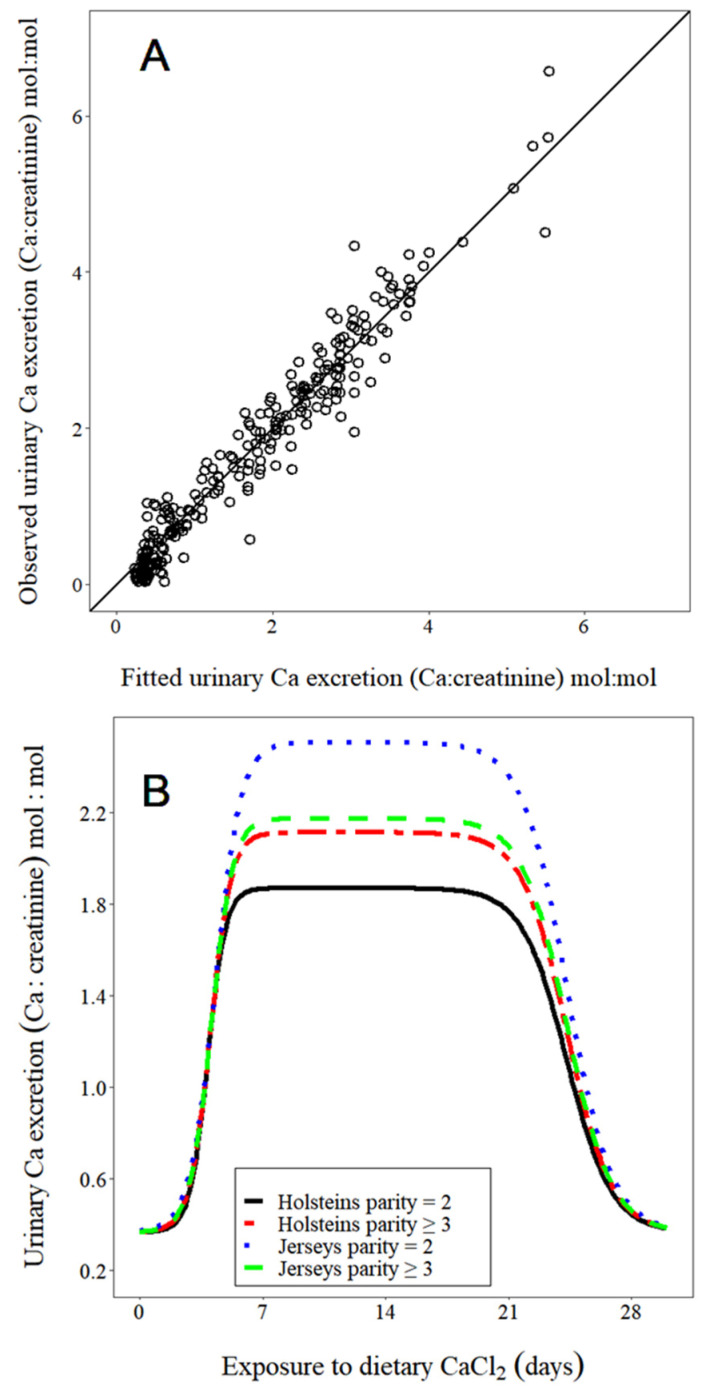
Modeled urinary Ca excretion (expressed as Ca:creatinine, mol:mol) in response to dietary CaCl_2_ during the close-up dry period. (**A**) plot of observed vs. predicted; model RMSE = 0.39 and (**B**) plot of Equation (1) from the text with parameter estimates provided in Table 1.

A plot of observed vs. predicted values of UCE (Figure 2A) showed good agreement between the data and the fitted model. The time course nature of the data as well as the effects of breed, parity group, and their interaction are depicted in a plot of modeled UCE vs. time at mean centered values of equivalents Cl/day (Figure 2B). To provide a quantitative perspective of the magnitude of the response over time, we computed the observed UCE relative to maximal UCE values at several different times. Values observed over 7–21 days were >93% of maximal values but had fallen to <5% of maximal values by 28 days for all combinations of breed and parity.

### 3.2. Whole Blood BHB Concentrations

**Figure 3:**
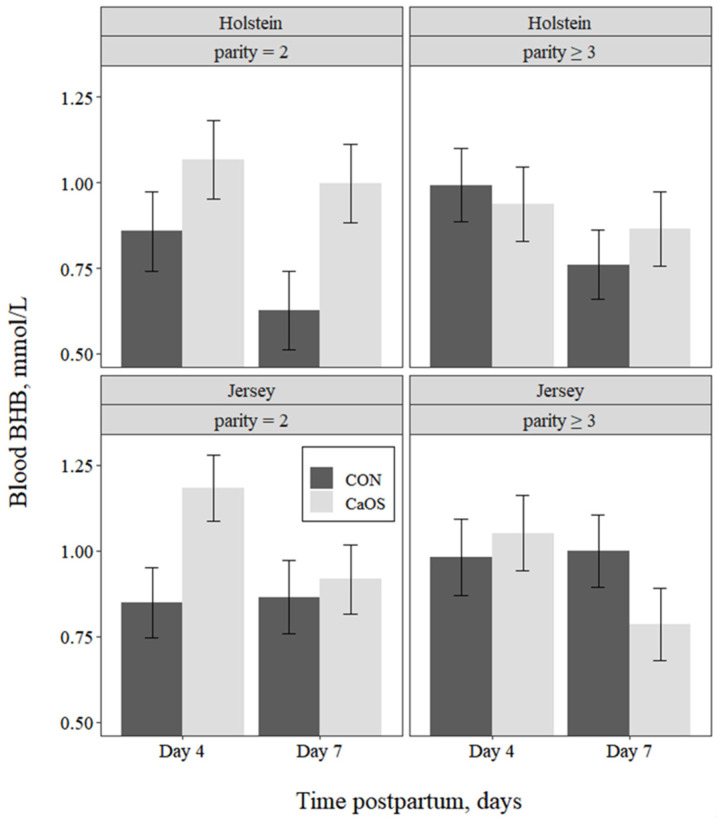
Least squares means for blood beta-hydroxybutyrate (BHB) concentrations by day postpartum, parity group, and breed in cows receiving either no intervention postpartum (CON) or 43 g of supplemental oral Ca bolus (Bovikalc, Boehringer Ingelheim, St. Joseph, MO, USA) within 3 h of parturition and again 12 h later (CaOS). Concentrations were affected by day postpartum (*p* = 0.028) and tended to be affected (*p* = 0.074) by the interaction of parity group with supplemental oral Ca (model RMSE = 0.23).

Whole blood BHB concentrations (Figure 3) decreased (*p* = 0.028) from day 4 to day 7 postpartum. In addition, averaged across days, there was a tendency for an interaction (*p* = 0.072) between parity and treatment. This occurred because BHB concentrations for CaOS vs. CON were numerically greater for parity = 2 (1.05 vs. 0.81 mmol/L) but were numerically lower for CaOS vs. CON for parity ≥ 3 (0.90 vs. 0.93 mmol/L).

### 3.3. Time Course Serum Ca Concentrations

**Figure 4:**
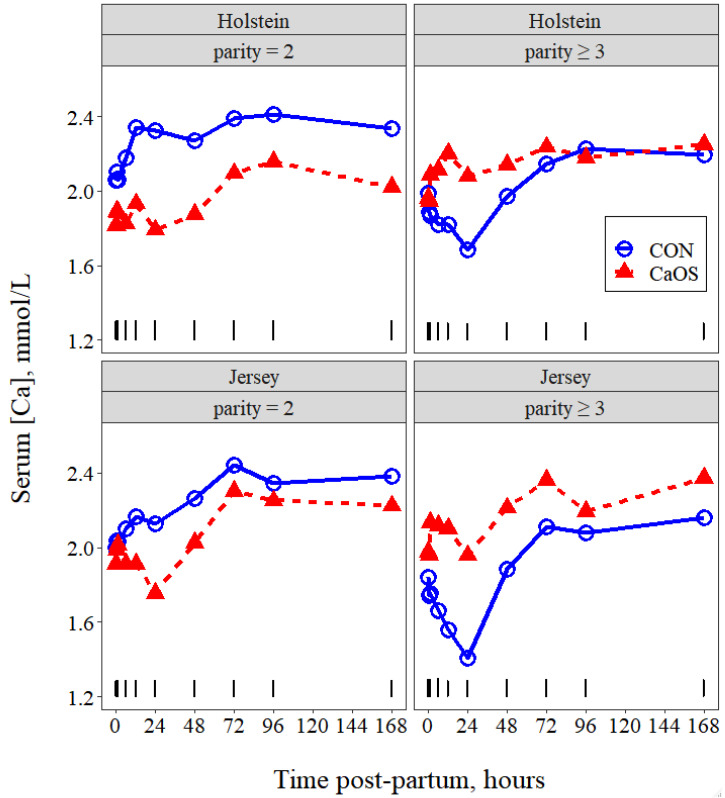
Least squares time course means of serum Ca concentrations postpartum in cows receiving either no intervention postpartum (CON) or 43 g of supplemental oral Ca bolus (Bovikalc, Boehringer Ingelheim, St. Joseph, MO, USA) within 3 h of parturition and again 12 h later (CaOS). Significant main effect of time (*p* = 0.016) and interactions (*p* ≤ 0.01) of time with breed, time with parity group, and time with parity group and supplemental oral Ca treatment; model RMSE = 0.17. Vertical bars above the *x*-axis represent the SE of breed × parity group × treatment × time point means.

Least squares means of serum Ca over time by breed, parity group, and treatment (Figure 4) revealed a significant effect of time (*p* = 0.016) and interactions (*p* ≤ 0.01) of time with breed, time with parity group, and time with parity group and treatment (time course plots of each of these least squares means are presented in Supplemental Figure S2). Based on evaluation of breed × time means and parity group × time means, we restricted our comparisons of these least squares means to the 12 and 24 h time points. For both breed and parity group, there were tendencies (*p* ≤ 0.10) for differences at each of these timepoints suggesting that serum Ca concentrations were lower for Jerseys vs. Holsteins and for parity ≥ 3 than parity = 2. Based on inspection of the parity group × treatment × time means, comparisons were made at each time point. A significant (*p* ≤ 0.01) interaction of treatment with parity group was found from 6 to 48 h, inclusive, because serum Ca numerically decreased for supplemental oral Ca treatment for parity = 2 but numerically increased for supplemental oral Ca for parity ≥ 3 at these times. Finally, for all combinations of breed, parity group, and treatment, serum Ca concentrations had increased well above Ca0 values by 72 h and based on single degrees of freedom contrasts for linear and quadratic effects, there was no change (*p* ≥ 0.7) beyond this time point.

### 3.4. Univariate Serum Ca Concentrations

**Figure 5:**
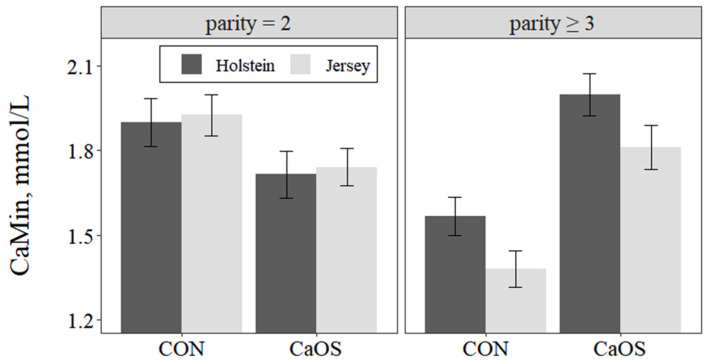
Least squares means for the minimum serum Ca concentration observed (CaMin) in cows receiving either no intervention postpartum (CON) or 43 g of supplemental oral Ca bolus (Bovikalc, Boehringer Ingelheim, St. Joseph, MO, USA) within 3 h of parturition and again 12 h later (CaOS). Significant effects observed due to parity group (*p* = 0.043), supplemental oral Ca treatment (*p* = 0.040), and the interaction of treatment with parity group (*p* < 0.001). There was a tendency (*p* = 0.077) for an interaction of breed with parity group. Model RMSE = 0.16.

Serum Ca concentration immediately postpartum (range = 1.90 to 1.95 mmol/L) was unaffected (*p* ≥ 0.5) by any categorical covariates. Minimum observed serum Ca concentrations (Figure 5) were affected (*p* ≤ 0.05) by parity group, treatment, and the interaction of parity group with treatment and tended to be affected (*p* = 0.08) by the interaction of parity group with breed. Because supplemental oral Ca caused a decrease in CaMin of 0.18 mmol/L for Parity = 2 but an increase of 0.43 mmol/L for parity ≥ 3, the overall effect of increasing parity group on CaMin was only −0.13 mmol/L. Therefore, to clarify the effects of breed and parity group in the absence of supplemental oral Ca, single degrees of freedom contrasts were constructed to evaluate the effects of breed, parity group, and their interaction for cows only in the CON group. These revealed that the decrease in CaMin due to parity group (*p* < 0.001) was 0.44 mmol/L and tended to be greater (interaction of parity group with breed, *p* = 0.09) for Jerseys (1.92 to 1.38 mmol/L) than for Holsteins (1.90 to 1.57 mmol/L).

**Figure 6:**
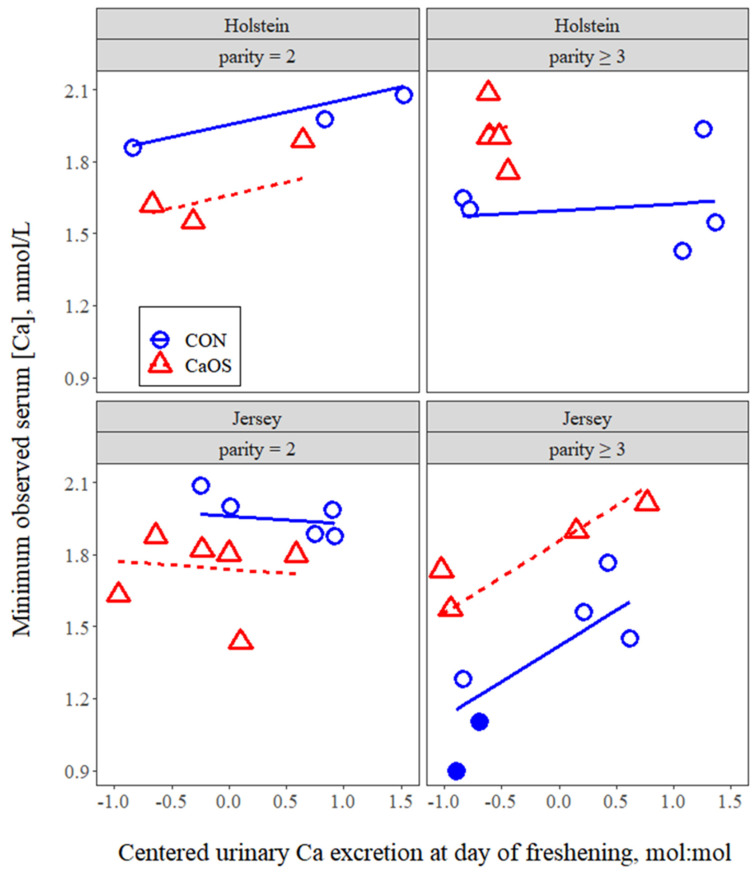
Minimum serum Ca concentration observed vs. centered urinary Ca excretion at day of calving (UCEdoc, expressed as Ca:creatinine ratio, mol:mol) in cows receiving either no intervention postpartum (CON) or 43 g of supplemental oral Ca bolus (Bovikalc, Boehringer Ingelheim, St. Joseph, MO, USA) within 3 h of parturition and again 12 h later (CaOS). Overall mean urinary Ca:creatinine ratio was 1.21, which corresponds to 0 on the *x*-axis. Interaction of UCEdoc with parity group and breed (*p* = 0.004) and slope of the line different from zero (*p* < 0.001) for parity ≥ 3 Jerseys. Two cows that contracted clinical milk fever are indicated in filled symbols. Model RMSE = 0.13.

The effects of continuous covariates on postpartum serum Ca concentrations are presented in Table 2. For each 1-unit increase in UpHdoc, Ca0 decreased (*p* = 0.028) by 0.12 mmol/L and for each 1-unit increase in UCEdoc, Ca0 increased (*p* = 0.031) by 0.10 mmol/L. For each of UpHdoc and UCEdoc, the nature of the response was consistent across all groups of cows; there were no interactions (*p* > 0.10) between either of these covariates with breed or parity group. For parity ≥ 3 Jerseys, Ca0 decreased 0.19 mmol/L with each 1-kg increase in colostrum yield (*p* = 0.002). Minimum serum Ca concentrations were unaffected by UpHdoc (*p* > 0.1) but were affected by the interaction of UCEdoc with parity group and breed (*p* = 0.004). The nature of this interaction is depicted in Figure 6, where the increase in CaMin with increasing UCEdoc for parity ≥ 3 Jerseys (0.293 mmol/L per 1-unit increase, *p* < 0.001), but a lack of change in other breed × parity combinations (*p* > 0.1) is evident.

**Figure 7:**
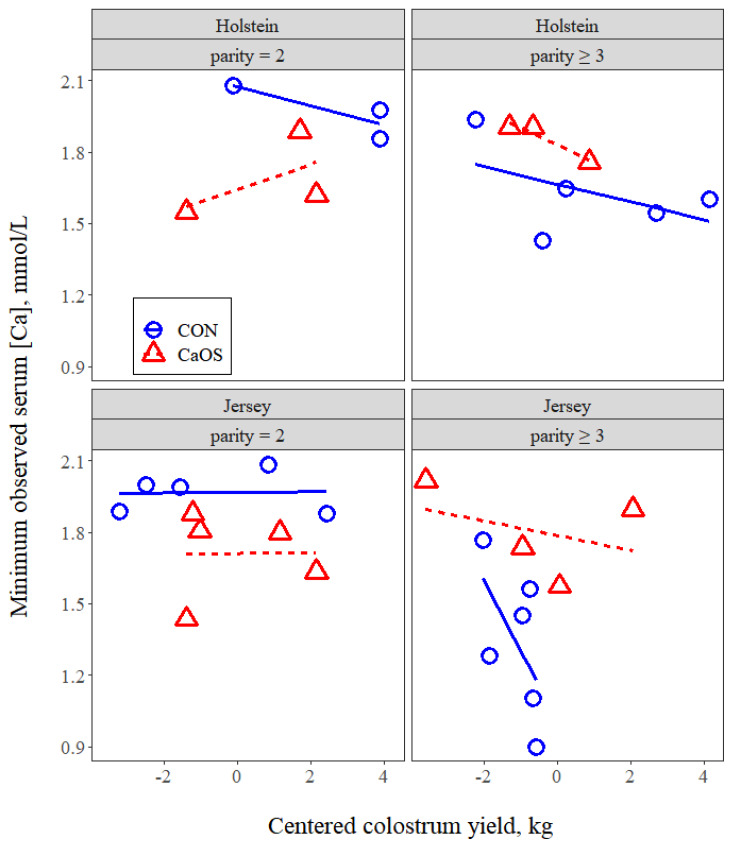
Minimum observed serum Ca concentration vs. centered colostrum yield (kg) in cows receiving either no intervention postpartum (CON) or 43 g of supplemental oral Ca bolus (Bovikalc, Boehringer Ingelheim, St. Joseph, MO, USA) within 3 h of parturition and again 12 h later (CaOS). Overall mean colostrum yield from all cows was 3.93 kg, which corresponds to the 0 on the *x*-axis. There was a tendency for an interaction of colostrum with parity group, breed, and treatment (*p* = 0.10). Slope of the line for parity ≥ 3 Jerseys in the CON group different from zero (*p* = 0.05). Model RMSE = 0.15.

Finally, there tended to be an interaction (*p* = 0.10) of colostrum yield with parity group, breed, and treatment. Concentrations of CaMin decreased 0.293 mmol/L per 1-kg increase in colostrum for parity ≥ 3 Jerseys in the CON group (*p* = 0.05) but were unaffected (*p* > 0.10) by colostrum yield for other combinations of parity group, breed, and treatment (Figure 7).

## 4. Discussion

### 4.1. General Approach

A simple approach to top-dress and mix by hand a commercially available food-grade solution of CaCl_2_ for group-housed cows fed a TMR was successful in acidifying close-up cows. Combined with routine monitoring of UpH and incrementally adjusting the CaCl_2_ dose, we maintained UpH within our desired range of 6 to 7 (overall mean was 6.32 ± 0.46). This represents an approach that could be implemented on small dairies, where separate mixing of a TMR solely for close-up dry cows is not feasible. Cows’ aversion to acidogenic salts and the importance of properly mixing the anion source within the TMR are well known [14]. Cows appeared to be fully acidified by the time the first urine sample was collected, which was 3 to 4 days after they were switched to the diet containing CaCl_2_ (Figure 1).

Intakes of Cl were 89% of delivered. Cows in this study appeared to be modestly successful in sorting against the CaCl_2_; however, based on urinary pH values, we judge this to be acceptable. We are not aware of other data that report the proportion of acidogenic salts offered that were actually consumed; thus, we are unable to compare our results to other studies.

### 4.2. Serum Ca Concentrations

Several important points are evident from previous reports of serum or plasma Ca concentrations with intensive sampling in the periparturient period. First, Ca concentrations obtained immediately postpartum do not represent a baseline value from which subsequent postpartum concentrations have fallen. Rather, they are already substantially lower than samples taken ≥1 day prepartum and reflect the drain of colostrogenesis [23,29,30]. Second, if samples are obtained several days postpartum, Ca concentrations return to prepartum baseline levels [16,23,29]. Third, postpartum nadir concentrations are typically observed 12–24 h postpartum [21,23,30]. The above observations represent data obtained from Holsteins [21,23,30], Jerseys [29], and Holstein-Jersey crosses [16]. We are unaware of any previous single study reporting an evaluation of either breed or any interactions among parity, breed, and oral Ca treatment for multiparous cows.

The results of the current study were broadly consistent with previously published patterns. First, nadir serum Ca concentrations were observed at 24 h. Second, serum Ca concentrations had returned at least to Ca0 levels by 48 h, and, by 72 h, they had stabilized at levels well above Ca0, which likely reflected prepartum baseline levels. These data underscore the fact that the critical window of vulnerability to milk fever is generally only within the first 48 h postpartum. Third, both cases of clinical milk fever in the current study were observed in fourth parity Jerseys; this is consistent with well-known principles that increasing parity and Jerseys vs. Holsteins are risk factors for clinical milk fever. Moreover, the timing of the diagnoses (one case at 12 h and the other at 24 h post-calving) corresponded to typical timing of nadir Ca concentrations. Fourth, decreasing minimum serum Ca concentrations within increasing parity, more so for Jerseys than Holsteins, is consistent with known associations between hypocalcemia and breed or parity.

### 4.3. Whole Blood BHB Concentrations

The overall prevalence of hyperketonemia (defined as blood BHB ≥ 1.2 mmol/L) was 16% (10/64) among all samples obtained. This was well below averages of 22% [31] or 24% [32], each of which represent large-scale studies from a wide variety of countries. Overall, these data indicate that cows in this trial were below the typical risk for hyperketonemia and that negative effects of high blood BHB concentrations did not inordinately affect Ca homeostasis.

### 4.4. Urinary Acidification and Ca Excretion

The maximum change for both UpH (Figure 1) and UCE (Figure 3) was essentially reached within one week. The maximum rate of decline of urinary calcium excretion was observed at 27 days following introduction of CaCl_2_. This may reflect adaptation to dietary acidogenic salts, whereby bone Ca mobilization was shutting off, and the cows were using other means of compensating for dietary acidogenicity. The mean value of UCEdoc in this study was 42.8 ± 30.3% (mean ± SD) of maxium, indicating both that cows were generally far along on the downward trend by day of calving and that there was substantial variation among cows.

The range of estimated urinary pH values at day of calving obtained in this study (5.49–7.80) indicate that a broad range of acidification of cows was achieved. With each 1-unit decrease in estimated urinary pH at day of calving, serum Ca concentration immediately postpartum significantly increased by 0.12 mmol/L, but the increase in minimum observed serum Ca concentration (0.072 mmol/L) was numerically much smaller and was not significantly different from zero. Thus, increasing dietary acidogenicity in this study did help mitigate the Ca drain due to colostrogenesis (i.e., Ca0) but did not mitigate the subsequent and larger demands of lactogenesis (i.e., CaMin).

Inspection of data from previous reports of the effect of dietary acidogenic salts on DM intake indicate a linear relationship. Computing the slopes of relationships obtained (i.e., kg DM intake decrease per 1-unit decrease in urinary pH) resulted in values of 0.78 [11], 0.27 [15], 0.27 [12], and 0.50 [33]. The linear nature of these responses indicates that application of any amount of dietary acidogenicity comes at the expense of some depression in DM intake and underscore the risk of excessive acidification. Thus, the goal is to strike a balance between adequate acidification without excessive depression of DM intake.

Wide variation in UCEdoc among cows (mean = 1.21 ± 0.78 SD) and an associated increase in Ca0 (0.10 mmol/L per 1-unit increase in UCEdoc) were observed. As for mean Ca0 values, neither UCEdoc nor the relationship between Ca0 and UCEdoc were affected by breed, parity group, or their interaction (*p* ≥ 0.23). Combined, these data suggest that prepartum Ca mobilization, and the mitigating effect of prepartum Ca mobilization on serum Ca immediately postpartum were similar for Holsteins vs. Jerseys and among parity groups. However, there was an increase in minimum observed serum Ca in response to increased Ca mobilization (0.29 mmol/L per 1-unit increase in UCEdoc) only in parity ≥ 3 Jerseys. These data indicate that, when Ca mobilization was low, older Jerseys experienced a lower nadir serum Ca and had increased risk for hypocalcemia (Figure 6). This perhaps reflects a reduction in bone PTH receptors with increasing age and the lower number of intestinal vitamin D receptors in Jerseys vs. Holsteins [34]. It is also important to note that minimum observed serum Ca was not increased as urinary pH decreased. Thus, these data highlight the fact that the benefit of dietary acidogenic salts is not through the induced acidosis (e.g., reduced urinary pH) per se but rather is mediated through the consequent effects on Ca mobilization.

Standard practice is 21 days of exposure to dietary acidogenic salts before calving, which corresponds to the typical time cows spend in the close-up dry cow pen. However, the optimal length of time is unknown. It does not take long for cows to benefit from dietary acidogenic salts; maximal urinary Ca excretion occurred within one day of introducing HCl [35] or one week of introducing a blend of acidogenic salts [36,37]. Three recent studies investigated some aspects of extended exposure to dietary acidogenic salts. Two studies [38,39] reported no differences in urinary pH, serum Ca, DM intake, or milk yield over the range of 21 to 42 days. However, Lopera et al. [33] reported that 42 vs. 21 days had some negative consequences (gestation length was reduced by two days, milk yield was reduced by 2.5 kg/day, and pregnancy per AI was reduced). There is an additional report [40] evaluating length of exposure to prepartum transition diets, but this included not only the introduction of acidogenic salts but also changing the provision of forage, concentrates, minerals, and provision of monensin rather than evaluating the isolated effect of diet acidogenicity. The results of our current study suggested unfavorable associations between increasing time of exposure to dietary acidogenic salts and both UpH (Figure 1) and UCE (Figure 2B). It is possible that a shorter exposure might be optimal for mitigating hypocalcemia. Uncertainty regarding the actual time of parturition poses a practical challenge in applying reduced time of prepartum dietary acidogenic salts. In addition, potential benefits of shorter exposure would need to be balanced against the potentially negative effects of moving cows [41], particularly if some cows are moved very close to actual calving. These considerations require more rigorous evaluation in prospective studies.

### 4.5. Colostrum Yield and Hypocalcemia

Average colostrum yields were higher (*p* = 0.018) for Holsteins (5.0 kg) than Jerseys (3.3 kg). These yields were a bit lower than other recent reports for Holsteins of 6.4–6.8 kg [38,42] or Jerseys of 4.1–4.3 kg [43,44]. The loss of Ca in colostrum by dairy cows is normally about nine times the plasma Ca pool [45] and is believed to occur faster than it can be replaced from either dietary supply or bone reserves [46]. To our knowledge, this is the first report to indicate that increasing colostrum yield is a risk factor for hypocalcemia. Colostrum yield was reported to not differ between clinically hypocalcemic cows and normal cows for Finnish and Finnish-Ayrshire crosses [47] or without a description of breed [48]. Megahed et al. [30] found no relationship between colostrum yield and serum plasma Ca concentrations 1d postpartum in Holsteins. No information was offered on the potential effects of parity in these reports. We found that increased colostrum yield was associated with decreases in serum Ca concentrations immediately postpartum for parity ≥ 3 Jerseys and also nadir serum Ca for parity ≥ 3 Jerseys in treatment CON. Colostrum production was not affected (*p* ≥ 0.23) by either parity group or the interaction of parity group with breed. Therefore, the relationships we observed between serum Ca concentrations and colostrum were not explained by increased colostrum yield per se. Because Ca can return to the circulation from the mammary gland and this return increases with increasing intramammary pressure [9], partial milking of colostrum or udder insufflation may represent mitigation strategies for milk fever in older cows [49]. Alternatively, when colostrum yields are high in older cows, this could be used as an indicator for targeted milk fever prevention strategies.

### 4.6. Oral Ca Supplementation

Minimum serum Ca observed was decreased by 0.18 mmol/L by supplemental oral Ca for parity = 2 but increased by 0.43 mmol/L for parity ≥ 3 cows by supplemental oral Ca. Similarly, there were significant interactions between supplemental oral Ca and parity group on serum Ca concentrations from 6 to 48 h postpartum. The mean serum Ca response over these times due to supplemental oral Ca was −0.29 mmol/L for parity = 2 and +0.40 mmol/L for parity ≥ 3 cows. Response to supplemental oral Ca was consistent for Holsteins vs. Jerseys as there were no interactions with breed (*p* > 0.10). One study [20] found no interaction of oral calcium supplementation with parity group (2, 3, or ≥4) for production or health outcomes but did not evaluate blood Ca as a response variable. We found no other published data allowing for evaluation of an interaction of supplemental oral Ca with parity for serum or plasma Ca for multiparous cows. Reports consisted either of third and greater parity cows [16,23] did not include a term corresponding to the interaction of parity with supplemental oral Ca [17,18,21,22,50], or did not state if second parity cows were in the experiment [17]. Both the magnitude and the duration of the increase in serum Ca obtained from supplemental oral Ca in parity ≥ 3 cows in the current study was substantially greater than these latter reports. This finding could be at least partially explained if supplemental oral Ca similarly reduced serum Ca in parity = 2 cows in those reports, which pooled data for multiparous cows across parity.

The direction of both the serum Ca and blood BHB responses to supplemental oral Ca in parity = 2 cows was unexpected. Reductions in Ca and increases in BHB can be associated with negative outcomes but also can be a result of increased milk production in healthy cows. One report noted that oral Ca supplementation increased herd removal for parity = 2 but not for parity ≥ 3 cows [21]. In addition, an interaction of parity group with supplemental oral Ca was reported whereby differential effects on blood acid-base parameters and disease incidence were found on primiparous vs. multiparous cows [17]; potential differences between second parity vs. third and greater parity cows were not evaluated in this study. Clearly, the potential differential effects of supplemental oral Ca based on parity warrants further investigation.

Because the supplemental oral Ca sources employed typically are themselves acidogenic salts (i.e., CaCl_2_, CaSO_4_), they also have direct effects of acidification as evidenced by their effects on urinary pH [16,22,23]. Thus, it is important that cows receiving supplemental oral Ca not be over-acidified by the implementation of excessively low DCAD diets prior to calving. Reporting either urinary pH or blood gas data just before and just after calving in experiments involving dietary acidification and oral Ca supplementation would allow for evaluation of this effect.

## 5. Conclusions

Topdressing and hand mixing a commercially available food grade solution of CaCl_2_ offers a practical approach to acidification of close-up dry cows for small dairies unable to mix a separate TMR for a small group of cows that are close to calving. The current practice of 21 days of acidification may be excessive for optimal effectiveness; reducing the time of acidification may enhance the effectiveness of this technology without the risk of reducing DM intake that accompanies increasing the dose of dietary anions. Further research, designed to specifically evaluate a reduced time of prepartum exposure to acidogenic salts, is warranted. Reduced urinary Ca excretion prepartum and increased colostrum yield appear to be independent risk factors for hypocalcemia in older cows, particularly Jerseys. Differential effects of oral Ca supplementation based on parity group warrant further investigation.

## Figures and Tables

**Table 1:** Model parameters for describing urinary Ca excretion, expressed as Ca:creatinine ratio (mol:mol) ^1^.

Parameter	Estimate	SE	*p*-Value
BL, mol Ca:mol creatinine	0.368	0.068	
A, mol Ca:mol creatinine	1.80	0.35	
Covariate			
Breed	0.698	0.303	0.011
Parity group	−0.089	0.297	0.38
Breed × parity group	0.576	0.293	0.025
Centered mean equivalents of Cl/day	0.605	0.215	0.002
λ_1_, days	2.83	0.42	
μ_1_, days^−1^	0.738	0.099	
λ_2_, days	26.8	1.88	
μ_2_, days^−1^	0.197	0.050	
Model RMSE	0.39		

^1^ Model parameters correspond to an ascending and then descending logistic equation (Equation (1) in the text), where BL = baseline value, A = maximum increase above baseline, μ_1_ and μ_2_ are maximum rates of increase and decrease (days^−1^), respectively and λ_1_ and λ_2_ are the times (days) corresponding to these maximum rates. For the categorical covariate of Breed, Holstein had a coefficient of 0 and Jersey had a coefficient of 1 and for parity group, parity = 2 cows had a coefficient of 0 and parity ≥ 3 cows had a coefficient of 1. Mean equivalents/day calculated during the time of dietary CaCl_2_ addition.

**Table 2:** Slopes of the relationships between univariate measurements of serum Ca (mmol/L) and a one-unit change in centered continuous covariates ^1^.

	Holsteins				Jerseys			
	Parity = 2		Parity ≥ 3		Parity = 2		Parity ≥ 3	
Covariate	CON	CaOS	CON	CaOS	CON	CaOS	CON	CaOS
Serum Ca immediately postpartum (Ca0) ^2^								
UpHdoc ^a^	−0.120 ± 0. 052 ^c^		−0.120 ± 0. 052 ^c^		−0.120 ± 0. 052 ^c^		−0.120 ± 0. 052 ^c^	
UCEdoc, mol:mol ^a^	0.100 ± 0. 044 ^c^		0.100 ± 0. 044 ^c^		0.100 ± 0. 044 ^c^		0.100 ± 0. 044 ^c^	
Colostrum, kg	0.034 ± 0. 036		−0.049 ± 0. 030		0.015 ± 0. 029		−0.190 ± 0. 055 ^c^	
Minimum serum Ca (CaMin) ^3^								
UpHdoc	−0.0722 ± 0.049	−0.0722 ± 0.049	−0.0722 ± 0.049	−0.0722 ±0.049	−0.0722 ± 0.049	−0.0722 ± 0.049	−0.0722 ± 0.049	−0.0722 ± 0.049
UCEdoc, mol:mol ^b^	0.115 ± 0.0753	0.115 ± 0.0753	0.0412 ± 0.0624	0.0412 ± 0.0624	−0.0482 ±0.0878	−0.0482 ±0.0878	0.293 ± 0.0716 ^c^	0.293 ± 0.0716 ^c^
Colostrum, kg	−0.040 ± 0.062	0.0524 ± 0.0742	−0.0374 ± 0.040	−0.0729 ± 0.128	0.0016 ± 0.0428	0.0016 ± 0.0627	−0.293 ± 0.142 ^c^	−0.0309 ± 0.050

^1^ CON = Control group (no intervention post-partum) and CaOS = 43 g supplemental oral Ca (Bovikalc, Boehringer Ingelheim, St. Joseph, MO, USA) within 3 h of parturition and again after 12 h. UpHdoc is the estimated urinary pH at day of calving and UCEdoc is estimated urinary Ca excretion at day of calving, expressed as Ca:creatinine (mol:mol). ^2^ Serum Ca concentration determined within 3 h postpartum. Because no terms for serum Ca immediately postpartum involving supplemental oral Ca were significant (*p* ≥ 0.37) and these data were collected before supplemental oral Ca was administered, means were computed by breed and parity group. ^3^ Minimum serum Ca concentration observed over 0–168 h postpartum. ^a^ Covariate main effect (*p* ≤ 0.05).^b^ Interaction of covariate with parity group and breed (*p* = 0.004). ^c^ Individual slope different from zero (*p* ≤ 0.05).

## Data Availability

Data are available upon request from the authors.

## Supplementary Materials

The following are available online at https://www.mdpi.com/article/10.3390/ani11113131/s1, Table S1: Allotment of cows to experimental treatments by breed and parity group. Table S2: Ingredient and nutrient composition of prepartum diets. Figure S1: Spaghetti plots of prepartum urinary Ca excretion (expressed as Ca:creatinine ratio, mol:mol) over time of exposure to dietary CaCl_2_. Figure S2: Least squares time course means of serum Ca concentrations postpartum in cows receiving either no intervention postpartum (CON) or 43 g of supplemental oral Ca bolus (Bovikalc, Boehringer Ingelheim, St. Joseph, MO, USA) within 3 h of parturition and again 12 h later (CaOS). (**A**) Breed × time least squares means; tendency for significant differences (*p* ≤ 0.10) at 12 and 24 h. (**B**) Parity group × time least squares means; tendency for significant differences (*p* ≤ 0.10) at 12 and 24 h. (**C**) Parity group × treatment × time least squares means; treatment × parity group interaction (*p* ≤ 0.01) at 6–48 h. Time is treated as a categorical variable to facilitate viewing of the early time points. In each panel, the vertical bars above the *x*-axis represent SE of group × time means.
