# Supplementary material for: Effects of Postpartum Supplemental Oral Ca for Dairy Cows Fed Prepartum Dietary Acidogenic Salts
Source: Animals (Basel). 2021 Nov 2;11(11):3131. doi: 10.3390/ani11113131 (PMC8614558; doi:10.3390/ani11113131)
Supplement: Supplementary file 1 [file animals-11-03131-s001.zip › animals-1403546-supplementary.pdf]

Supplemental Table S1. Allotment of cows to experimental treatments by breed and parity group.

Group	CON	CaOS
	Holstein	
parity = 2	n=3	n=3
parity ≥ 3	n=5	n=4
	Jersey	
parity = 2	n=5	n=6
parity ≥ 3	n=6	n=4

CON = Control group (no post-partum intervention);. CaOS = 43 g supplemental oral Ca (Bovikalc, Boehringer Ingelheim, St. Joseph, MO) within 3 hours of parturition and again after 12 hours.

Supplemental Table S2. Ingredient and nutrient composition of prepartum diets.

Ingredient	% of DM
Wheat hay	29.1%
Alfalfa hay	14.5%
Grass silage	26.7%
Wet brewers grain	4.7%
Grain mix	25.1%
Analyzed Nutrients[1]	Concentration
DM (% as-is)	45.0 ±1.0
CP (% DM)	13.49 ±0.26
ADF (% DM)	34.87 ±0.79
aNDF (% DM)	48.23 ±0.58
EE (% DM)	3.21 ±0.13
Ash (% DM)	9.67 ±1.21
NFC (% DM)	25.4 ±1.23
Ca (% DM)	0.66 ±0.03
P (% DM)	0.34 ±0.01
Mg (% DM)	0.37 ±0.02
K (% DM)	1.26 ±0.07
S (% DM)	0.21 ±0.01
Na (% DM)	0.29 ±0.02
Cl (% DM)	0.65 ±0.06
DCAD[2] meq/kg DM (mEq/kg DM)	134 ±20
Predicted composition	
NE_L[3] (Mcal/kg DM)	1.45
ME[3] (Mcal/kg DM)	2.27
Vitamin A (KIU/kg DM)	4.14
Vitamin D (KIU/kg DM)	1.06
Vitamin E (IU/kg)	88.1
Monensin (ppm)	14

[1]Weekly samples (n=18) were dried and ground; then 9 composites of biweekly samples were analyzed. [2]Dietary Cation Anion Difference = [(Na % of DM/0.023) + (K % of DM/0.039)] – [(S % of DM/0.016) + (Cl % of DM/0.0355)]. [3]Net Energy of lactation (NE_L) and metabolizable energy (ME) predicted by the Cornell Net Carbohydrate and Protein System (v 6.5, Cornell University, Ithaca, NY).

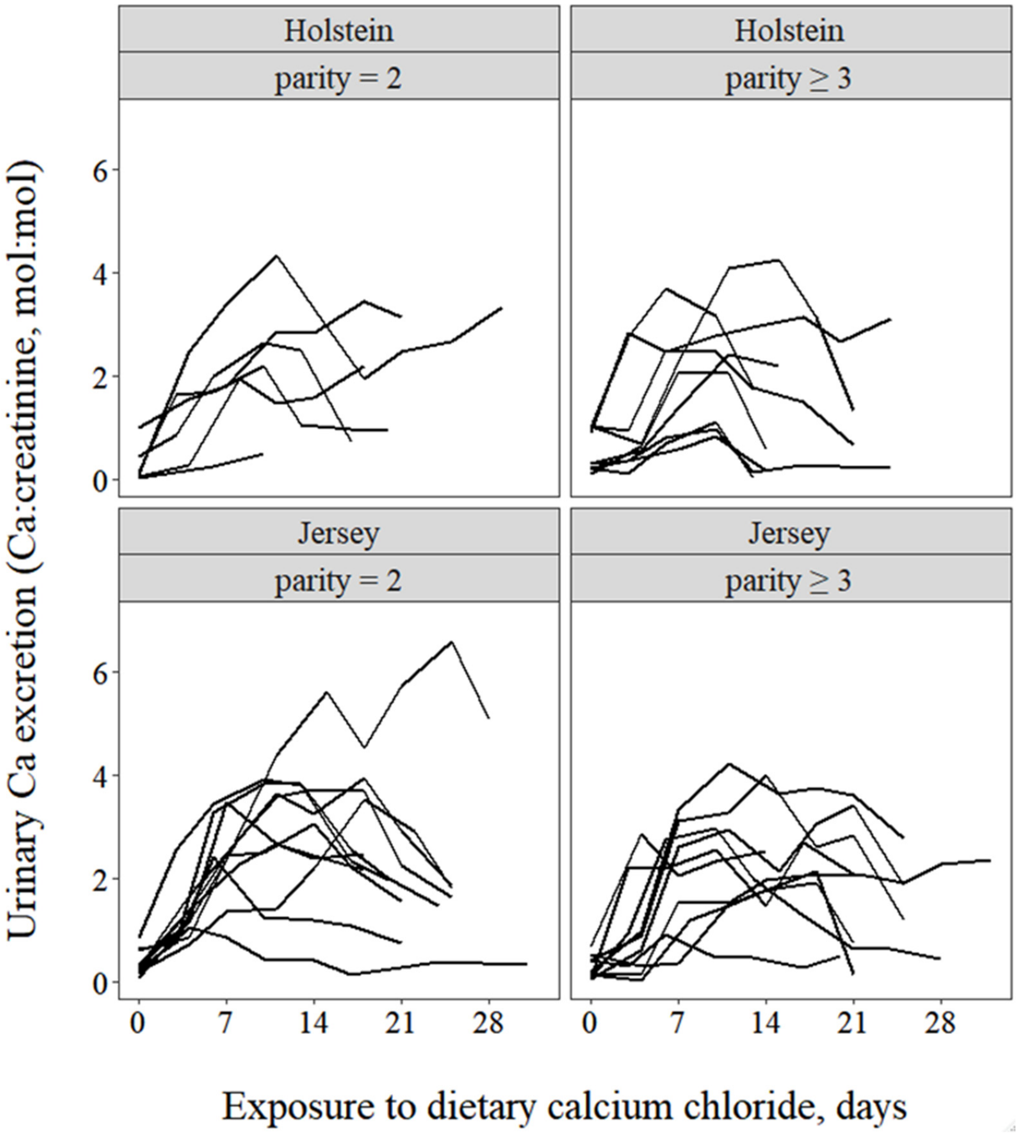

Supplemental Figure S1. Spaghetti plots of prepartum urinary Ca excretion (expressed as Ca:creatinine ratio, mol:mol) over time of exposure to dietary $CaCl_2$.

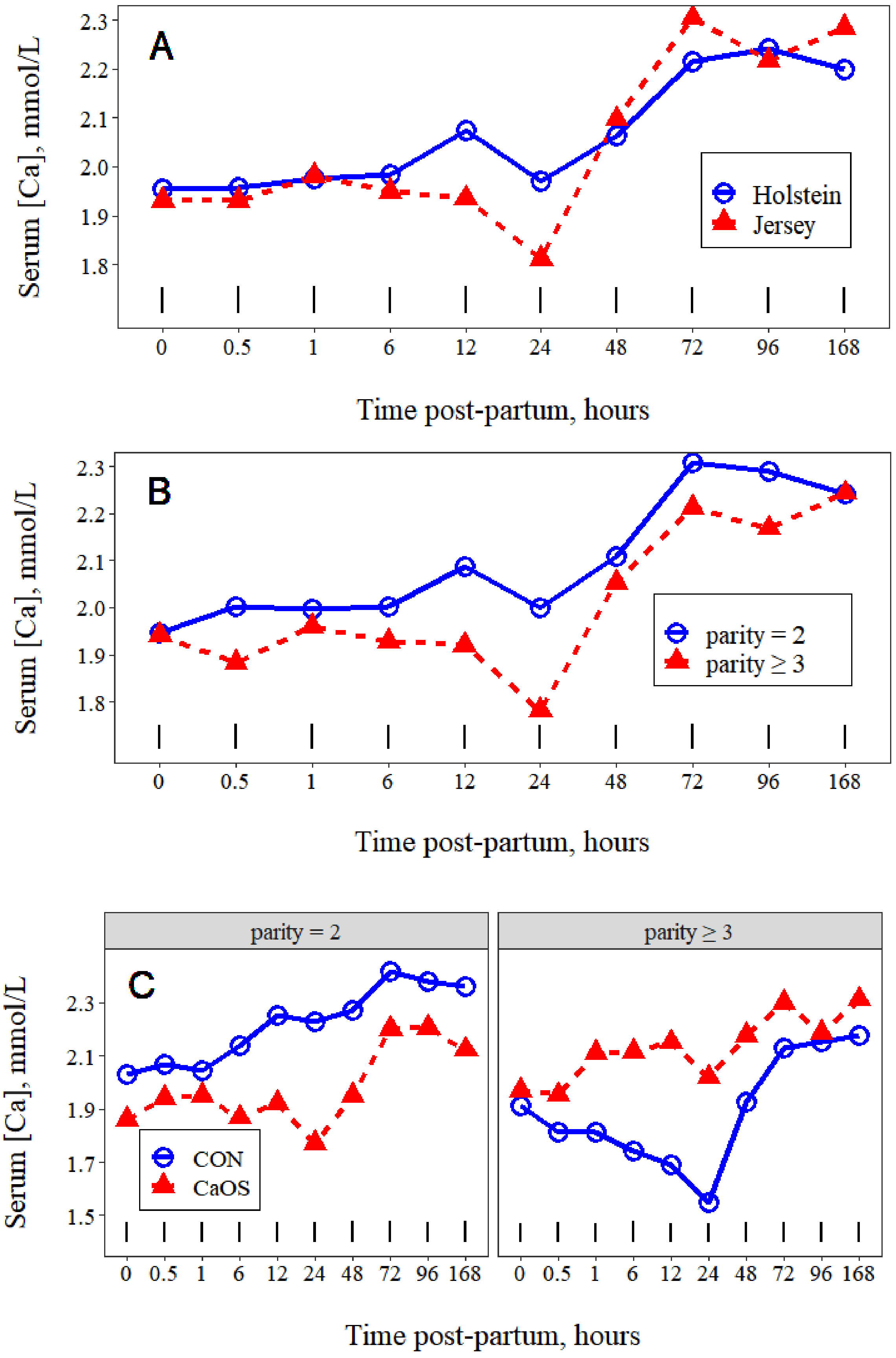
A
Holstein
Jersey
Serum [Ca], mmol/L
2.3
2.2
2.1
2.0
1.9
1.8
0
0.5
1
6
12
24
48
72
96
168
Time post-partum, hours
B
parity = 2
parity ≥ 3
Serum [Ca], mmol/L
Time post-partum, hours
C
parity = 2
parity ≥ 3
CON
CaOS
Serum [Ca], mmol/L
2.3
2.1
1.9
1.7
1.5
Time post-partum, hours

Supplemental Figure S2. Least squares time course means of serum Ca concentrations postpartum in cows receiving either no intervention postpartum (CON) or 43 g of supplemental oral Ca bolus (Bovikalc, Boehringer Ingelheim, St. Joseph, MO) within 3 hours of parturition and again 12 hours later (CaOS). (A) Breed x time least squares means; tendency for significant differences ($p \leq 0.10$) at 12 and 24h. (B) Parity group x time least squares means; tendency for significant differences ($p \leq 0.10$) at 12 and 24h. (C) Parity group x treatment x time least squares means; treatment x parity group interaction ($p \leq 0.01$) at 6-48 hours. Time is treated as a categorical variable to facilitate viewing of the early time points. In each panel, the vertical bars above the *x*-axis represent SE of group x time means.
